# Supplementary material for: Asynchronous Embryo Transfer Followed by Comparative Transcriptomic Analysis of Conceptus Membranes and Endometrium Identifies Processes Important to the Establishment of Equine Pregnancy
Source: Int J Mol Sci. 2020 Apr 7;21(7):2562. doi: 10.3390/ijms21072562 (PMC7177982; doi:10.3390/ijms21072562)
Supplement: Supplementary file 1 [file ijms-21-02562-s001.zip › Supplementary Table 1.docx]

**Supplementary Table 1** Equine primer pair details: gene symbol, primer sequence, annealing temperature, product size and accession number for all genes used for qRT-PCR analysis.

| Gene symbol | Ta (^o^C) | Product size (bp) | Forward sequence | Reverse sequence | Accession no. |
| --- | --- | --- | --- | --- | --- |
| LIFR | 58 | 280 | GTTTCCTTAATTCCAGACACTC | CAACGTAGCATCTAATTCCC | XM_023625722.1 |
| LIF | 65 | 264 | GGAGTTGTGCCCCTGCTGCTA | CGTGGGAAAGGGCGGGAAGTC | XM_023646870.1;  XM_023646869.1;  XM_023646868.1;  XM_023646867.1;  XM_023646866.1 |
| PTGFR | 58 | 185 | CTTAGCCCTTGTCGTTTCA | TGTTGGCCATTGTCACC | NM_001081806.3;  XM_005610256.3; XM_023640208.1;  XM_014739993.2;  XM_023640207.1 |
| INSR | 62 | 101 | CGAGTTGGATTATTGCCTCAAAG | CGTACTCACTCTGATTGTGCTTCTG | XM_023644612.1;  XM_023644611.1;  XM_023644610.1;  XM_023644609.1;  XM_023644608.1;  XM_023644607.1 |
| IL6ST | 62 | 259 | GCACTGTTGATTATTCTCCTG | GTTGAAGCATCTTTGGTCCT | NM_001301172.2;  XM_023625263.1;  XM_005604257.3;  XM_005604255.3;  XM_023625262.1 |
| IGF1 | 60 | 137 | ACGCTCTTCAGTTCGTGTGT | CAGCCTCCTCAGATCACAGC | XM_005606472.3;  XM_005606471.3;  XM_005606470.3;  XM_005606469.3;  NM_001082498.2 |
| OXTR | 62 | 218 | CATCGTGCTGGCCTTCATCGTGTG | GGTAGCCGGAGGAGCAGCAGAGGA | XM_014731360.2;  XM_023620041.1;  XM_023620040.1 |
| NDN | 58 | 222 | CGGTAGTTACAAGAAATGGTG | CGACCCTTCACATAGATGAG | XM_001492612.4 |
| H19 | 58 | 224 | GTCTCTGTTCCTTTACTTTCC | GTTCCTTTAGCTTAACCTTCC | NR_027326.1 |
| SNRPN | 60 | 109 | GATCTCAGCAACAGCAAATACC | CTACCTGGATGTTCCAATACTG | XM_005602853.1; XM_005602852.1 ; XM_005602849.1; XM_005602848.1; XM_005602847.1; XM_005602850.1; XM_005602851.1 |
| PEG10 | 62 | 216 | AGAGTTTACCCACCCATGAG | GAGTAATCAGGCCATCTTTCAC | NM_001172559.2 |
| SLC2A1 | 63 | 308 | CACGGGCTTCGTGCCCATGT | GGGTCACGTCTGCCGTTCCG | NM_001163971.2;  XM_005607003.3 |
| SLC1A4 | 63 | 365 | TGTGTGGCCGCGGTGTTCAT | GGCGACGTCTCCTCCTCCGA | XM_001493515.3 |
| SLC1A5 | 60 | 104 | TCAGCCTGCCGGTTCACGAC | TCCTGCCCCAAAGGCGTCAC | XM_001917363.1 |
| SLC2A3 | 58 | 233 | CCAGGAGATGAAAGATGAGAG | ATAGTATTAACCACACCCGCA | XM_001498757.1 |
| SLC2A5 | 63 | 281 | CCGATTAAGAAGGAAGGGAG | TTTCTGCCAAGTCTATTCACC | NM_001081877.1 |
| SLC5A1 | 60 | 104 | TCAGCCTGCCGGTTCACGAC | TCCTGCCCCAAAGGCGTCAC | XM_001917363.1 |
| SLC7A5 | 60 | 297 | GAAAGGTGACATCTCCAATCTG | GTGATAGTTCCCGAAGTCCA | XM_001916639.4 |
| SLC38A2 | 62 | 377 | CAGCCTGACACAACCAGCGGC | GGGTGACAGCCACTAACACAGCC | XM_001489523.3 |
| SLC43A2 | 60 | 181 | CATCCACTCTGCCGTCGGGG | REV 5' GCCCAGCAGCCCCACATTCA | XM_001502290.1 |
| GAPDH | 53 | 112 | AGGCCATCACCATCTTCCAG | CCAGCCTTCTCCAAGGTAGT | NM_001163856.1 |
| HPRT1 | 58 | 232 | GAGATGTGATGAAGGAGATGG | CTTTCCAGTTAAAGTTGAGAGG | XM_001490189.2 |
| SRP14 | 55 | 101 | CTGAAGAAGTATGACGGTCG | CCATCAGTAGCTCTCAACAG | XM_001503583.2 |
